# Synergies between radiotherapy and immunotherapy: a systematic review from mechanism to clinical application

**DOI:** 10.3389/fimmu.2025.1554499

**Published:** 2025-08-11

**Authors:** JingLong Jiang, HouZe Li, QingCui Ma, Jing Liu, Fu Ren, YingQiu Song, TianLu Wang, KeYan Li, Ning Li

**Affiliations:** ^1^ Department of Pathology and Pathophysiology, Shenyang Medical College, Shenyang, China; ^2^ The Sixth Affiliated Hospital of Xinjiang Medical University, Wulumuq, China; ^3^ Liaoning University of Traditional Chinese Medicine, Shenyang, Liaoning, China; ^4^ Department of Biochemistry and Molecular Biology, Shenyang Medical College, Shenyang, China; ^5^ Shenyang Key Laboratory for Phenomics, Shenyang Medical College, Shenyang, China; ^6^ Department of Anatomy, College of Basic Medical Sciences, Shenyang Medical College, Shenyang, China; ^7^ Department of Radiotherapy, Cancer Hospital of Dalian University of Technology (Liaoning Cancer Hospital & Institute), Shenyang, China; ^8^ Faculty of Medicine, Dalian University of Technology, Dalian, China; ^9^ Department of Radiotherapy, Cancer Hospital of China Medical University, Liaoning, China; ^10^ Department of Cardiology, First Affiliated Hospital of Jinzhou Medical University, Jinzhou, China; ^11^ Liaoning Province Key Laboratory for Phenomics of Human Ethnic Specificity and Critical Illness (LPKL-PHESCI), Shenyang Medical College, Shenyang, China

**Keywords:** radiotherapy, immunotherapy, PD-L1, immune checkpoint inhibitors, tumor

## Abstract

The combination of radiation therapy (radiotherapy) with immunotherapy is changing the landscape of cancer treatment. Radiation damage tumor cell DNA through high-energy rays directly causes cell death or growth arrest; Immunotherapy works by boosting a patient’s own immune system’s ability to recognize and destroy cancer cells. The combination of the two not only enhances the local control of the tumor, but may also activate the systemic anti-tumor immune response, transforming the “cold” tumor into a “hot” tumor, thereby improving the survival rate and quality of life of patients. In recent years, this combined treatment approach has shown remarkable efficacy in a variety of cancers, especially non-small cell lung cancer and melanoma, among others. However, how to optimize radiation dose, timing and combination with immunotherapy drugs remains the focus of research. This paper first discusses the effect of radiotherapy on immune system, then analyzes the killing effect of radiotherapy and its mechanism, and finally discusses the latest progress and challenges of the combined application of radiotherapy and immunotherapy. Each section aims to provide clinicians and researchers with an in-depth understanding with a view to optimizing treatment strategies and improving outcomes for cancer patients.

## 1 Introduction

The combined application of radiation therapy (radiotherapy) and immunotherapy represents an important breakthrough in the field of cancer treatment. Radiotherapy not only exerts an anti-tumor effect by directly killing tumor cells, but also activates a systemic anti-tumor immune response by releasing tumor-associated antigens, reshaping the tumor microenvironment, and enhancing immune cell infiltration. Immunotherapy, especially immune checkpoint inhibitors (such as PD-1/PD-L1 and CTLA-4 inhibitors), further enhances the efficacy of radiotherapy by lifting the tumor’s suppression of the immune system. While preclinical studies overwhelmingly support the synergy between radiotherapy and immunotherapy, clinical translation remains inconsistent. Heterogeneous patient responses, lack of standardized protocols, and dynamic immune modulation pose significant challenges. This article will systematically review the synergies of radiotherapy and immunotherapy from basic mechanisms to clinical applications, and explore their potential and challenges in multiple cancer types.

## 2 Effects of radiation therapy on the immune system

### 2.1 Radiotherapy induces tumor cell death and antigen release

Radiation therapy can trigger a series of immunostimulating and immunosuppressive events, including cell death, production of cytokines and chemokines in the tumor microenvironment, release of tumor antigens and endogenous danger molecules, activation of antigen-presenting cells, recruitment of growth factors and interleukins, and chemotactic signaling that induces chemotaxis of bone marrow-derived cells. These events trigger a complex set of immune responses that are important for the combination of radiation therapy and immunotherapy (1). Radiation therapy can increase the ability of tumor cells to release antigens, making them easier to recognize by the immune system. DNA damage caused by radiation therapy may cause tumor cells to release neoantigens, increasing the immune system’s response to the tumor (2). Radiation therapy can increase the expression of MHC molecules on the surface of tumor cells, making them more easily recognized by cytotoxic T cells (3). Radiation therapy in cancer treatment is not only a method to directly kill tumor cells, but also capable of triggering a series of complex immune stimulation and immunosuppressive events. These events are of great significance for the combined application of radiotherapy and immunotherapy, and provide new ideas for the development of more effective cancer treatments.

### 2.2 Activation of immune cells by radiation therapy

Tumor-associated macrophages (TAMs) can have a positive or negative impact on tumor growth, invasion, and metastasis. TAMs are usually abundant in tumor tissue, and it has been confirmed that certain subgroups of TAMs can strongly affect tumor progression and interfere with almost all types of cancer treatment (4). Radiation therapy induces a wide range of antitumor effects by inducing tumor cells to release microparticles (RT-MPs), and causes immune cell death mainly through ferroptosis. After phagocytosis by tumor-associated macrophages (TAMs), these RT-MPs can promote the polarization of TAMs from M2 type to M1 type and regulate the anti-tumor interaction between TAMs and tumor cells (5). In a mouse model, neoadjuvant low-dose gamma-radiation was found to modulate the function of macrophages to an anti-tumor mode, showing neither immunosuppressive nor pro-angiogenic activity, and producing T-cell-attracted chemokines (6).

**Figure 1:**
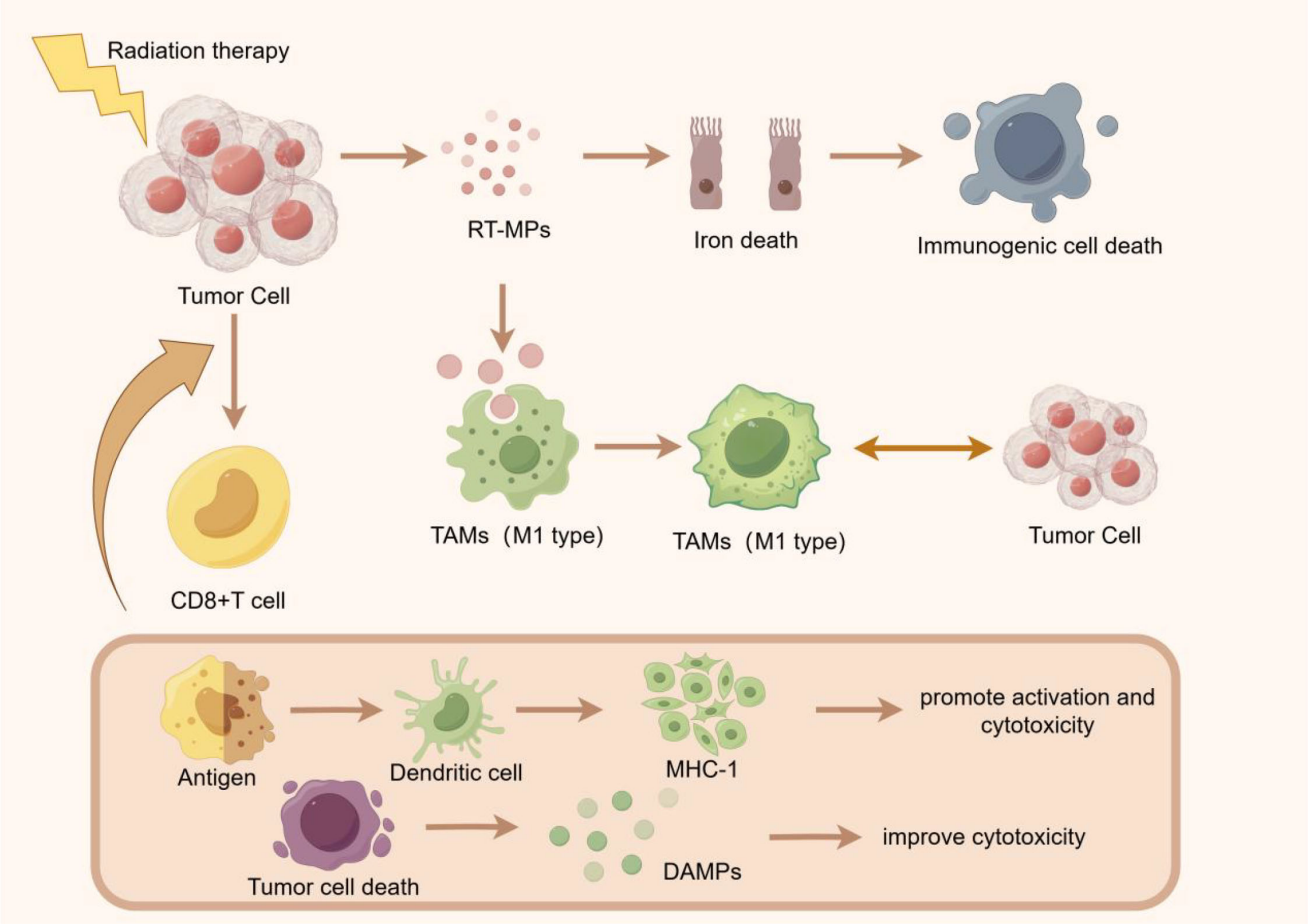
Radiation therapy initiates its anti-tumor effects by inducing the release of radiation-induced membrane permeability alteration particles (RTMPs) from tumor cells, triggering ferroptosis and subsequent immunogenic cell death. This process facilitates the polarization of tumor-associated macrophages (TAMs) from the immunosuppressive M2 phenotype to the immunostimulatory M1 phenotype, thereby enhancing anti-tumor immunity. Concurrently, tumor cell death releases tumor-associated antigens and damage-associated molecular patterns (DAMPs), which are captured by dendritic cells and presented to CD8+ T cells via major histocompatibility complex class I (MHC-I) molecules. This antigen presentation cascade activates cytotoxic T lymphocytes, augmenting the immune system’s capacity to recognize and eliminate tumor cells.

Radiation therapy can enhance the expression of mutation-related neoantigens on tumor cells and enhance the killing effect of the immune system on tumors by increasing the expression level of neoantigens (7).Radiotherapy enhances the anti-tumor immune response by inducing the activity of CD8+T cells and the death of tumor cells. Radiotherapy induced tumor cell death releases DAMage-associated molecular patterns (DAMPs), which in turn promote the activation and killing of CD8+T cells (8)Radiation therapy can promote antigen (9)recognition, activate dendritic cells, and stimulate the expression of MHC-1 molecules, thereby improving the killing effect of CD8+T cells on tumors (**Figure 1**).

NLR is considered to be an indicator of inflammation and immune status, and can be used to predict the subsequent development and severity of many diseases, including infections, inflammatory diseases and tumors. Higher NLR values are generally associated with more severe diseases and poor prognosis (10). NLR values increase significantly after radiation therapy, and high NLR values are associated with lower overall survival in patients. This suggests that the ratio of neutrophil to lymphocyte may reflect the inflammatory response and immune status after radiotherapy, and thus affect the treatment effect and survival rate of patients (11).

IDO is an immunomodulator related to tryptophan metabolism. Its activity works primarily by regulating inflammation and T cell tolerance. IDO activity can be triggered in a variety of ways, including innate immune responses and T cell activation. In the tumor microenvironment, IDO plays an important role in the response to apoptotic cells and its influence on the function of Treg cells (12). The sentence structure follows standard medical writing conventions while accurately conveying the original Chinese meaning. The parenthetical clarification about Kyn/Trp ratio measurement maintains readability without disrupting the flow (13).

### 2.3 Regulation of tumor microenvironment by radiation therapy

Radiation therapy can reshape the immune environment in tumors in a number of ways. First, radiotherapy can induce the death of tumor cells, release pro-inflammatory (and anti-inflammatory) mediators, increase the immunostimulant (and immunosuppressive) cells infiltrated by tumors, and enhance neoantigen expression. Secondly, radiotherapy can also alter the immunogenicity and immunosubsidiarity of tumors by modulating the interaction of immune cells and other cell types in the tumor microenvironment (14). Immunostimulatory signals generated by radiation therapy help to increase tumor antigen presentation and effector T cell activation, while immunosuppressive signals hinder radiation-induced tumor rejection. Recent studies have shown that small extracellular vesicles (exosomes) play an important role in radiation-induced remodeling of the tumor immune microenvironment. Exosomes can carry immunomodulatory molecules and tumor antigens and transmit messages between tumor tissue and immune cells, thereby influencing tumor immune response (15). Radiotherapy disrupts immune cell infiltration and induces a new inflammatory response in the tumor immune microenvironment (TIME). In theory, radiotherapy may be more beneficial for patients with “Cold” TIME because it may damage existing CD8+ T cells (16). Tumor cell death induced by radiation therapy is not only a physical process, but also a biological “signaling” process. With the death and lysis of tumor cells, a large number of pro-inflammatory and anti-inflammatory mediators are released into the tumor microenvironment. These mediators not only change the local immune status of the tumor, but also attract more immune cells to infiltrate into the tumor tissue. Moreover, both the number and function of exosomes released by tumor cells and immune cells changed, and these changes further affected the direction and intensity of tumor immune response.

Radiotherapy has immunostimulating effect on local tumor control. It can improve the effectiveness of immunotherapy by enhancing the immune response and altering the tumor microenvironment. This local effect helps control tumor growth and metastasis, and ultimately improves patient survival (17). Radiation therapy and immunotherapy have a synergistic effect, probably because they produce a local immune-stimulating effect. In animal experiments, the researchers found that tumor-carrying mice given PD-L1 inhibitors and radiation at the same time had a stronger immune activation effect compared to other treatment timing. This suggests that giving immunotherapy and radiation at the same time can enhance immune activation (18).

The distant effect refers to the invasion and proliferation of immune cells triggered by radiation-induced immune cell death and antigen release during radiation therapy, which leads to the shrinkage and prevention of metastasis of tumors far from the area of radiation therapy. The occurrence of the distant effect is related to the radiation dose. Lower doses of radiation may lead to the development of immune tolerance, while higher doses of radiation can trigger antigen presentation and immune cell death, thus triggering the distant effect. However, tumors also suppress the immune response by promoting infiltration of regulatory T cells (Tregs) and M2 macrophages, as well as up-regulation of immune checkpoints. Therefore, combining immunotherapy with radiotherapy can prolong the anti-tumor immune response within the tumor and enhance the suppressive effect of the tumor by simultaneously targeting the immune checkpoint and the tumor-promoting cells (19). This can be achieved by increasing antigen expression and release of immunostimulatory factors in the tumor microenvironment through radiation therapy. This in turn recruits and activates antigen-presenting cells (APCs), which initiate an anticancer immune response when they migrate to the tumor draining lymph nodes and cross-activate CD8+ cytotoxic T lymphocytes (CTLs) (20). The distancing effect during radiotherapy reveals the complex interaction between radiation and the immune system, which opens up new avenues for tumor treatment. By modulating the dose of radiotherapy, we can use the death of immune cells and the release of antigens to trigger an anti-tumor response of the immune system, not only directly attacking tumors in the area of radiotherapy, but also producing a suppressive effect on tumors far away from the area of radiotherapy. Combination therapy, which combines immunotherapy with radiotherapy, not only prolongs the anti-tumor immune response within the tumor, but also enhances the therapeutic effect by simultaneously targeting immune checkpoints and promoting the death of tumor cells.

## 3 The synergistic effect of radiotherapy and immunotherapy

### 3.1 Mechanism of immune checkpoint inhibitors

Immune checkpoint inhibitors are a class of drugs used in cancer treatment. Their mechanism of action is to activate the body’s immune system to attack tumor cells by disactivating inhibitory signals in the immune system. Under normal circumstances, immune checkpoint molecules play an important role in regulating the immune response, helping to prevent the immune system from over-attacking its own tissues. However, tumor cells can use these immune checkpoints to evade immune system attack, thereby promoting tumor growth and spread (21).

Immune checkpoint inhibitors primarily act on two immune checkpoint molecules: PD-1 and CTLA-4. These drugs are able to block the function of immune checkpoint molecules in tumor cells or tumor microenvironment, allowing immune cells to regain their ability to attack tumors (21).

In addition to PD-1/PD-L1 and CTLA-4, there are other important immune checkpoint molecules such as LAG-3, TIM-3, TIGIT, etc., which also play a key role in tumor immune escape. LAG-3 inhibits T cell activity by binding to MHC Class II molecules, while TIM-3 interacts with its ligand Galectin-9 to cause T cell depletion (22, 23). TIGIT prevents T cells from receiving the necessary co-stimulatory signals through competitive binding to CD155 (24). Inhibitors that target these molecules, such as Relatlimab (a LAG-3 antibody), have been tested in certain types of cancer and have shown additional efficacy when combined with existing immune checkpoint inhibitors. For example, in a Phase II clinical trial in melanoma, Relatlimab in combination with Nivolumab significantly increased the objective response rate compared to Nivolumab alone (25). In addition, while TIM-3 and TIGIT inhibitors are mostly still in clinical trials, there is evidence that they could be an important part of future cancer treatment, either as monotherapeutics or in combination with other immune checkpoint inhibitors. Combining radiation therapy with these novel checkpoint inhibitors holds the promise of further enhancing the anti-tumor immune response, while focusing on side effect management and individualized treatment strategies.

### 3.2 Combination of radiotherapy and immune checkpoint inhibitors

**Figure 2:**
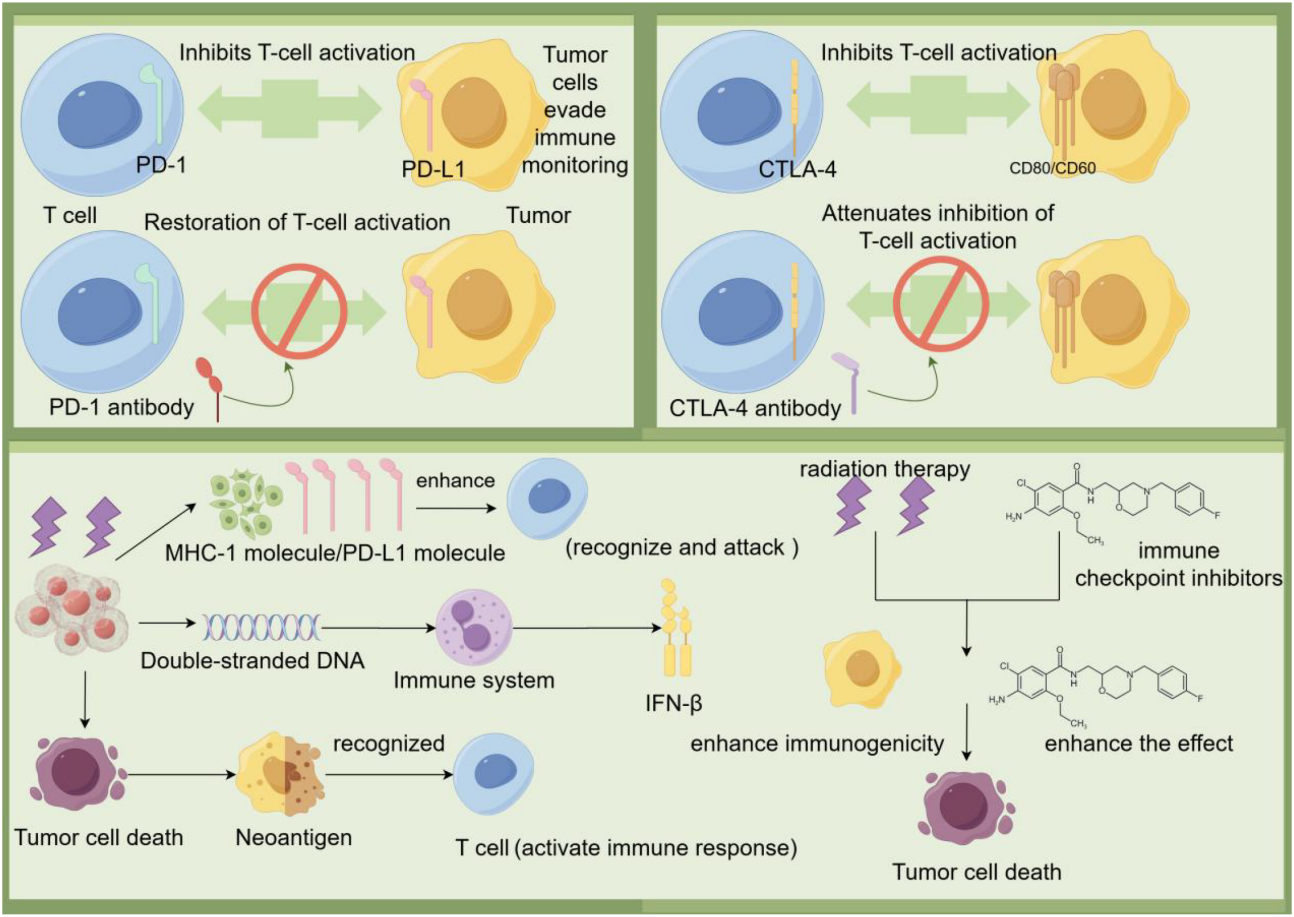
Tumor cells bind to PD-1 on T cells by PD-L1 on the surface, as well as inhibit T cell activation by binding to CTLA-4 on T cells by CD80/CD86, allowing tumor cells to evade immune surveillance. The use of PD-1 antibodies and CTLA-4 antibodies can block these inhibitory signals and restore T cell activation, which in turn enhances the immune response to the tumor. In addition, radiation therapy can cause tumor cell death and release double-stranded DNA and neoantigens, which are recognized by the immune system and activate T cells. Activated T cells enhance immunogenicity by releasing IFN-β, which promotes tumor cell death. The combination of immune checkpoint inhibitors and radiotherapy can further enhance the immune system’s ability to recognize and attack tumors, improving treatment effectiveness.

The mechanism by which immune checkpoint inhibitors act on lung cancer mainly involves the regulation of two immune checkpoints, PD-1/PD-L1 and CTLA-4. PD-1 is an immune checkpoint expressed on the surface of T cells and its ligands are PD-L1 and PD-L2. When PD-1 binds to PD-L1 on the surface of tumor cells, the activation of T cells and their ability to kill tumor cells will be inhibited, which allows tumor cells to cleverly evade the monitoring and clearance of the immune system. And PD-1 antibody and PD-L1 antibody can accurately block the binding between PD-1 and PD-L1, and then restore the activation state of T cells, and re-endow them with the ability to kill tumor cells. CTLA-4 is another important immune checkpoint that binds to CD80 and CD86 on the surface of tumor cells and inhibits T cell activation. CTLA-4 competitively binds CD80 and CD86 with CD28, thereby inhibiting T cell activation. By using CTLA-4 antibodies, it is possible to block the binding of CTLA-4 to CD80/CD86, weakening the inhibition of T cell activation and enhancing the role of the immune system against lung cancer (26). According to the study, it can be found that the combination of radiotherapy and CTLA-4 blocker can significantly improve the disease control rate of lung cancer patients. Radiation therapy can induce tumor cells to release double-stranded DNA, which in turn activates the immune system to produce interferon-β (IFN-β), thereby enhancing the immune response. Compared with immune checkpoint inhibitors alone, the combination of radiation therapy and immune checkpoint inhibitors improves disease control and survival rates in lung cancer patients. In conclusion, the combined application of radiation therapy and immune checkpoint inhibitors can improve the therapeutic efficacy of lung cancer by enhancing the immune response and inhibiting the immune escape mechanism of tumor cells. This provides a new therapeutic strategy for lung cancer patients (27). Immunotherapy drugs called immune checkpoint inhibitors, particularly those targeting PD-1/PD-L1 and CTLA-4, have led a ground-breaking revolution in lung cancer treatment. By reactivating suppressed T cells, these inhibitors are able to unlock the powerful potential of the immune system, enabling it to recognize and attack tumor cells. When radiotherapy is used in combination with immune checkpoint inhibitors, the synergy between them not only enhances the anti-tumor activity of the immune system, but also inhibits immune escape of tumor cells through different mechanisms. This combined treatment strategy not only improved disease control rates in lung cancer patients, but also significantly extended their survival (**Figure 2**).

Although radiotherapy combined with immune checkpoint inhibitors has shown significant efficacy in the treatment of lung cancer, this combination can also carry a range of side effects, especially immune-related adverse events (irAEs). Common irAEs include but are not limited to immune pneumonia, hepatitis, colitis, etc. (28). According to clinical research data, about 10%-20% of patients with non-small cell lung cancer who receive PD-1/PD-L1 inhibitors combined with radiotherapy may experience varying degrees of immune pneumonia, ranging in severity from mild to life-threatening (29). In addition, immune hepatitis occurs in about 5%-10% of patients, while immune colitis is relatively rare, but can equally cause serious health problems in some cases. For example, in a clinical trial involving the use of PD-1 antibodies in combination with radiotherapy to treat patients with advanced non-small cell lung cancer, several cases of immune pneumonia requiring glucocorticoid therapy were observed (30). This suggests that close monitoring of patients’ lung function and other relevant indicators is critical when implementing such combination therapies.

Radiation therapy can produce immunosuppressive effects by upregulating the expression of immune checkpoint molecules, but by combining with immune checkpoint inhibitors, it can change the immune microenvironment of liver cancer and restore anti-tumor activity (31). Studies have shown that performed in a mouse model of liver cancer, a single dose of 10 Gy radiation therapy is given, followed by four anti-PD-L1 injections every 3 days after radiation therapy. These results suggest that tumor growth was significantly inhibited in the combination treatment group, along with a significant improvement in survival. (90% in the combination group, 30% in the radiotherapy group, and 0% in the anti-PD-L1 group) (31). In another study, anti-PD-1 antibody was given to a mouse liver cancer model and 30 Gy SBRT radiotherapy was simultaneously administered, and the results showed that the infiltration level of CD8+ T cells in tumor tissue was significantly increased, the development of tumor was significantly inhibited, and the survival rate was also improved (32). In another study, radiation therapy, anti-PD-L1, and a combination of the two were applied to subcutaneous and liver tumors in mice, and the results showed that the infiltration level of T cells in subcutaneous tumors was significantly increased in the combination treatment group, while the level of T cells was not increased in the anti-PD-L1 group, and radiation therapy alone could not regulate the number of T cells. In addition, the combination treatment group showed longer survival and shrinkage of subcutaneous and liver tumors (33). A study evaluating the effects of radiation therapy combined with anti-PD-1 antibodies on unirradiated and irradiated tumors in a mouse model of liver cancer showed a significantly enhanced distant effect of both irradiated and unirradiated tumors in the combination treatment group and a higher level of activated CD8+ T cell infiltration (34). The results of these preclinical studies and clinical trials suggest that the combination of radiation therapy with immune checkpoint inhibitors can significantly enhance the efficacy of liver cancer therapy and provide better survival and tumor control.

Similarly, in the treatment of liver cancer, the combination of radiation therapy with immune checkpoint inhibitors also carries certain risks. In addition to the immune pneumonia mentioned above, immune hepatitis is of particular concern in patients with liver cancer. Studies have shown that the incidence of immune hepatitis may be as high as 15% in liver cancer patients treated with anti-PD-L1 antibodies combined with radiotherapy, and its severity can increase over time (35). In addition, because the liver itself has a rich population of immune cells, special caution is needed when evaluating the safety and efficacy of combination therapy. One clinical trial in patients with liver cancer reported that approximately 7% of patients stopped treatment due to intolerable immune-mediated liver toxicity (36). This finding highlights the importance of considering individual differences when developing treatment plans and suggests the need for effective monitoring mechanisms to identify and address potential side effects early.

Head and neck cancer (HNC) is a complex group of malignant tumors, with common types including oral cancer, pharyngeal cancer, laryngeal cancer, etc. Although significant progress has been made in traditional treatments such as surgery, chemotherapy, and radiotherapy, the prognosis for patients with recurrent or metastatic cases remains poor (37). In recent years, with the deepened understanding of tumor immunology, immunotherapy—particularly the application of immune checkpoint inhibitors—has brought new hope to HNC treatment (38). Notably, the combination of radiotherapy and immunotherapy has demonstrated superior efficacy compared to single-modality therapy. Research indicates that in head and neck cancer, radiotherapy can promote the uptake and processing of antigens by dendritic cells (DCs), and enhance the recognition and cytotoxic activity of cytotoxic T lymphocytes (CTLs) against tumor cells (39). In a study on recurrent/metastatic head and neck squamous cell carcinoma (R/M HNSCC), the treatment regimen combining radiotherapy with PD-1 inhibitor and concurrent chemoradiotherapy has demonstrated an objective response rate (ORR) of 70.0% and a disease control rate (DCR) of 100%, indicating promising therapeutic efficacy. This finding provides a novel strategy for the management of R/M HNSCC (40). Although the combination of radiotherapy and immunotherapy has shown promising results in the management of recurrent/metastatic head and neck squamous cell carcinoma (R/M HNSCC), further research is needed to optimize treatment strategies and expand its applicability. A key area of investigation is the identification of reliable biomarkers—such as PD-L1 expression levels, tumor mutational burden (TMB), or specific immune-related gene signatures—that can predict response to combined therapy. These biomarkers may help guide patient selection and improve clinical outcomes.

Prostate cancer is one of the most commonly diagnosed malignant tumors in men and a leading cause of cancer-related death (41). In the treatment of metastatic prostate cancer, combination therapy may help induce immune cell-mediated clearance of distant metastatic foci, namely the “abscopal effect (42). Research shows that combining radiotherapy with immunotherapy significantly boosts the number of prostate-specific CD8^+^ T cells systemically, with the greatest accumulation in the prostate gland. Concurrent administration yields maximal T cell expansion, and 12 Gy has been identified as the optimal radiation dose for enhancing CD8^+^ T cell infiltration. Compared to immunotherapy alone, this combination increases T cell accumulation by approximately fourfold (43). The combination of radiotherapy and immunotherapy has demonstrated synergistic effects in the treatment of prostate cancer, offering a promising direction for improving outcomes in patients with metastatic disease. Future research should focus on optimizing treatment protocols to maximize the “abscopal effect”—the phenomenon in which localized radiation induces systemic anti-tumor immune responses, leading to the regression of non-irradiated metastatic lesions.

Glioma, particularly glioblastoma multiforme (GBM), is the most common primary brain tumor in adults and is known for its high invasiveness and poor prognosis (44). Despite treatment progress, GBM prognosis remains dismal, urging new strategies. Recurrent gliomas, labeled “cold tumors,” poorly respond to immunotherapy due to low mutational burden, scant T-cell infiltration, and strong immunosuppressive microenvironment (45). A study on glioblastoma (GB) investigated the combination of hypofractionated stereotactic radiotherapy (hFSRT) and the anti-PD-L1 agent durvalumab. The study found that this combination therapy was well-tolerated and showed promising efficacy, with disease control observed in some patients and potential abscopal effects noted (46). Although the combination of hypofractionated stereotactic radiotherapy (hFSRT) and anti-PD-L1 therapy has shown initial promise in the treatment of glioblastoma, with good tolerability and disease control, further research is needed to enhance its efficacy, particularly in overcoming the intrinsic immunosuppressive nature of GBM. Given that recurrent gliomas are often classified as “cold tumors” with low mutational burden and minimal T-cell infiltration, strategies aimed at converting these tumors into “hot tumors” are crucial for improving response rates to immunotherapy.

### 3.3 Side effects of radiation versus immunotherapy

**Figure 3:**
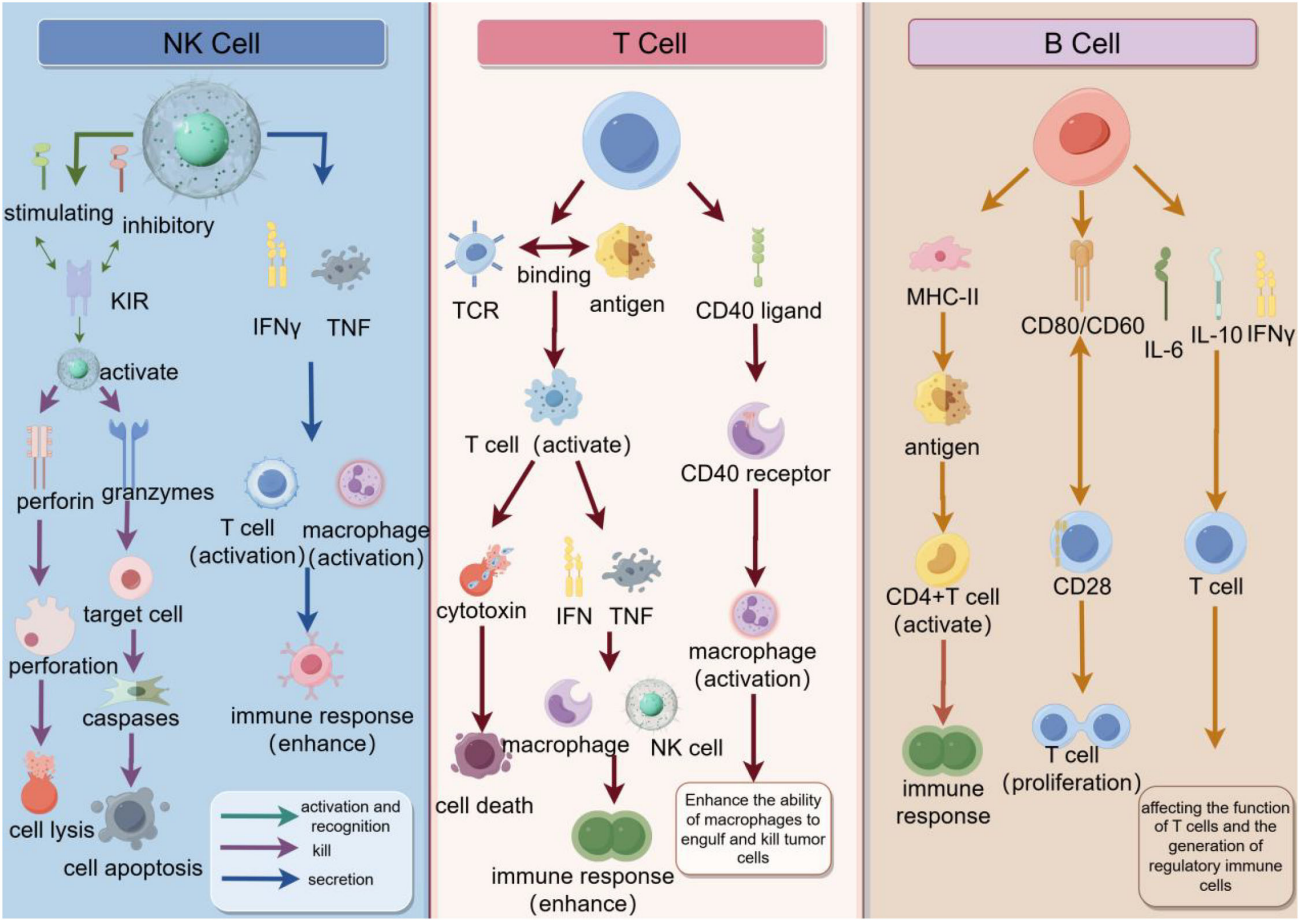
The synergy of natural killer cells (NK cells), T cells, and B cells in the immune response. NK cells recognize tumor cells through inhibitory and activator receptors on their surface and release perforin and granase to induce cell lysis and apoptosis, while enhancing the immune response with IFNγ and TNF. T cells recognize antigens through TCR and after activation secrete cytokines such as IFNγ and TNF, which can enhance the function of macrophages and NK cells, while interacting with B cells through CD40 ligands to promote antibody production. B cells recognize antigens through MHC-II and CD40/CD40 ligands, activate CD4+ T cells, and interact with T cells through CD28 to promote the proliferation and differentiation of B cells, and finally secrete antibodies such as IL-6, IL-10 and IFNγ to regulate the immune response. As a whole, these cells, through complex interactions and signaling, together constitute an efficient immune system to recognize and attack tumor cells.

The immune system recognizes and eliminates tumor cells by recognizing tumor-specific antigens or molecules induced by cellular stress. This process is called tumor immune surveillance (47). Radiotherapy enhances NK cell-mediated tumor killing through multiple mechanisms: Radiation upregulates stress ligands (MICA/B, ULBP) on tumor cells, augmenting NKG2D receptor recognition; Radiation-induced DNA damage triggers NK-activating DAMPs (HMGB1, ATP); Sublethal radiation doses (1–5 Gy) preserve NK cell viability while depleting immunosuppressive Tregs in tumor models. These effects are amplified when combined with CAR-NK therapies targeting tumor antigens (48, 49). T lymphocytes are able to recognize and bind to tumor-specific antigens through their T cell receptors (TCRS) and release cytotoxins to kill tumor cells. In addition, T lymphocytes are able to activate other immune cells, such as macrophages and natural killer cells, to enhance the immune response (47). Resident memory T cells (TRM), a memory subgroup of T lymphocytes, play an important role in solid tumors. TRM cells are mainly found in non-lymphoid tissues and express CD69 and CD103 integrins. They are found in a variety of tumors, including melanoma, lung cancer, urothelial cell carcinoma, and endometrial adenocarcinoma. Tumor tissues with a high density of CD8+ T cells in tumors are enriched with transcripts associated with tissue T cell residency, such as CD103 (50). B cells play a role by presenting antigens to T cells, interacting with T cell stimulatory receptors by expressing helper molecules, and producing cytokines (51). In summary, lymphocytes, as the central pillar of the immune system, play a vital role in the process of recognizing and eliminating tumor cells, and are involved in every step of tumor immune surveillance. They act as “hunters” of the immune system, using precise recognition and efficient attack to eliminate tumor cells that have been targeted by tumor-specific antigens or molecular markers induced by cellular stress (**Figure 3**).

Radiation therapy may lead to lymphocytopenia and the inability of immune cells to enter the tumor and its microenvironment effectively, thereby reducing the effectiveness of tumor control and thereby affecting the survival rate of patients (52). Wild et al. conducted a study on the survival outcomes of 101 patients with locally advanced pancreatic adenocarcinoma treated with radiotherapy and chemotherapy. The study found that lymphocyte counts below 500 mm³ and planned target volume were associated with poor overall survival. Krishna n et al., investigated the association between the severity of lymphocytopenia and spleen dose-volume parameters after chemoradiotherapy in 177 patients with locally advanced pancreatic adenocarcinoma. The study found that V5, V10, V15, and V20 of the spleen predicted lymphocytopenia severity and gave a mean dose limit of 9 Gy for the spleen. These studies suggest that radiation-induced lymphocytopenia may be closely associated with survival (53).

Lymphocytopenia after radiation therapy is associated with decreased survival in lung cancer patients. Lymphocytes play an important role in the immune response, playing a key role in fighting tumor cells and maintaining immune balance. Therefore, radiation-induced lymphocytopenia may impair a patient’s immune function and increase the risk of tumor recurrence and metastasis, thus affecting patient survival (54). The study found that the majority of patients with stage III non-small cell lung cancer who received maintenance immunotherapy experienced lymphocytopenia during radiation therapy, with 39 patients (59.0%) developing grade 3 or higher lymphocytopenia. At 3 months after radiotherapy, lymphocytes in 59 patients (89.3%) returned to normal, while seven patients (10.6%) still experienced persistent lymphocytopenia. Multivariate Cox regression analysis showed that recovery of lymphocytopenia was identified as a significant prognostic factor for progressive disease-free survival and overall survival (55).

In patients undergoing radiation therapy for breast cancer, lymphocyte counts reached their lowest point during treatment and gradually recovered in the month following treatment. Decreased lymphocytes may affect the function of the immune system and reduce the patient’s resistance to cancer, leading to a poor prognosis (56). Studies have found that radiation therapy led to significant reductions in circulating T and B cells, as well as a reduction in the number of bone marrow stem cells. Further experiments showed that transplantation of stem cells from bone marrow restored radiation-induced lymphocytopenia. These results suggest that radiation-induced lymphocytopenia has not only direct effects on circulating lymphocytes, but also indirect effects on stem cells and circulating lymphocytes in non-irradiated bone marrow (57). An XGboost model was developed using interpretable machine learning methods to predict radiation-induced lymphocytopenia in breast cancer patients. The researchers used feature sets such as clinical data, tumor features, blood cells, radiation and therapy as inputs to the model. They trained the model through a ten-fold cross-validation approach and evaluated the model’s predictions using measures such as sensitivity, specificity, accuracy, F1 score, ROC-AUC and PR-AUC. The results of the study showed that the XGboost model could predict radiation-induced lymphocytopenia well (58).

Palliative radiation therapy is a treatment used to relieve cancer-related symptoms, aiming to reduce symptoms such as pain, difficulty swallowing, difficulty breathing, bleeding, and tumor ulcers in patients (59). Studies have found that a subset of patients with advanced non-small cell lung cancer who received palliative radiation developed lymphocytopenia during and six to eight weeks after treatment. Lymphocyte counts dropped to 120 million/L and 80 million/L in the first and second weeks of treatment, respectively, but recovered somewhat after treatment ended, but remained below pre-treatment levels (60). Long-term non-intracranial radiation therapy may lead to severe and long-lasting lymphocytopenia. Moreover, radiation-induced lymphocytopenia is associated with reduced overall survival in patients treated with PD-1 inhibitors. These results suggest that for patients treated with PD-1 inhibitors, it may be more effective to opt for palliative radiation therapy because of less adverse effects on circulating lymphocytes (61).

Patients with severe RIL had a 65% increased risk of death compared to those with less severe lymphocytopenia. The study also found that patients with more severe RIL (Grade 4) had a 50% increased risk of death compared to patients with less severe lymphocytopenia (62). RIL is an independent poor prognostic factor for overall survival in patients with lung cancer. In lung cancer patients, radiation-induced lymphocytopenia has an adverse effect on overall survival, and the degree of lymphocytopenia can be mitigated by reducing exposure to stem cells and blood pools, thereby improving patient outcomes (63). The spleen plays an important role in regulating immune response. The findings suggest that low-dose areas of the spleen play an important role in reducing the number of lymphocytes. Thus, reducing the low-dose area of the spleen may be key to reducing the risk of radiation-induced lymphocytopenia (64). Radiation therapy is an important tool in cancer treatment, but the accompanying lymphopenia is a problem that cannot be ignored. The decrease of lymphocyte not only means the decline of immune function, but also is closely related to the survival rate of patients. The decrease of lymphocytes may prevent immune cells from entering the tumor and its microenvironment effectively, thus reducing the effectiveness of tumor control. This mechanism means that even if radiation therapy succeeds in shrinking the tumor, the patient may still be at risk of recurrence and metastasis due to the loss of lymphocytes.

## 4 Radiation therapy transforms “cold” tumors into “hot” tumors

Radiation therapy can promote the transformation of tumors from “cold” to “hot”. Radiation can activate the cGAS-STING signaling pathway. Radiation therapy promotes the release of double-stranded DNA (dsDNA) in the nucleus and the exposure of mitochondrial DNA (mtDNA), molecules that activate the cGAS-STING pathway and trigger a cascade of type I interferons. Type I interferons activate dendritic cells (DCS) to mature and present antigens to T lymphocytes, initiating a specific immune response (65, 66). Radiotherapy can stimulate tumor cells and stromal cells to release a variety of pro-inflammatory mediators and chemokines, such as CXCL9, CXCL10, CXCL11, etc., which can promote the infiltration of immune cells (such as DC, macrophages and T lymphocytes) (67). These changes work together to transform an immunosuppressive “cold” tumor into an immunoactive “hot” tumor, thereby improving the efficacy of immunotherapy.

### 4.1 Variations in the immunomodulatory effects of radiotherapy among different cancer types

The significant differences in the immune microenvironment among various types of tumors greatly influence the effectiveness of radiotherapy in transforming “cold” tumors into “hot” ones. This heterogeneity extends beyond just the tumor cells themselves and includes the surrounding stroma, vascular systems, and the composition and functional states of immune cells. Low-dose radiotherapy (LDRT) can modify immunosuppressive elements within the tumor microenvironment by regulating stromal components and enhancing the infiltration of immune effector cells. Specifically, LDRT can shift tumor-associated macrophages from an immunosuppressive M2 phenotype to a pro-inflammatory M1 phenotype, increasing the production of chemokines that recruit T cells and NK cells. Additionally, it downregulates key inhibitory cytokines such as TGF-β, thus fostering an environment that supports immune cell-mediated tumor attack (89). Compared to other tumor types, cancers such as pancreatic cancer demonstrate pronounced immune resistance. This is largely due to the presence of a dense desmoplastic stroma and minimal T cell infiltration, both of which hinder the penetration and efficacy of radiotherapy as well as various immunotherapies. The impact of high- versus low-dose radiotherapy on pancreatic cancer is primarily reflected in their distinct effects on the tumor microenvironment and immune response. High-dose radiotherapy can induce vascular damage within the tumor, reduce blood perfusion, and potentially enhance anti-tumor immunity through immunomodulatory mechanisms. In contrast, conventional low-dose fractionated radiotherapy may impair or suppress anti-tumor immune responses, limiting its potential to synergize with immunotherapeutic strategies (90). In conclusion, comprehending the variability in immune responses to radiotherapy among different tumor types is crucial for devising personalized therapeutic strategies. Selecting the appropriate radiation dose and combinational approaches based on the unique features of each tumor can more effectively facilitate the transition from a “cold” to a “hot” tumor microenvironment, thereby enhancing patient prognoses.

### 4.2 Optimization of treatment timing

The sequence of radiotherapy (RT) and immunotherapy significantly impacts treatment efficacy, as each modality dynamically reshapes the tumor immune microenvironment (TIME). Optimal timing can maximize synergistic effects while minimizing immune suppression.

Immunotherapy following radiotherapy can significantly improve the overall survival (OS) of patients with advanced lung cancer. The 6-month OS rate was 94.7% for patients who received immune checkpoint inhibitors (ICIs) after radiotherapy, compared to only 40.0% for those who received ICIs before radiotherapy (p < 0.001). Radiotherapy may activate anti-tumor immunity, but it can also increase PD-L1 expression, which suppresses this immune response. Combining ICIs with radiotherapy may help counteract this inhibition (68).

## 5 Radiotherapy dose and immunomodulation

Radiation therapy can activate the immune system through a variety of mechanisms and enhance the effect of immunotherapy (69). Radiotherapy can enhance the effect of immunotherapy by enhancing the expression of MHC I molecules, increasing the peptide library within tumor cells, and enhancing the expression of tumor-associated antigens (70). The dose and fractionation of radiotherapy can have an impact on immunotherapy. Low doses of radiotherapy can alter the tumor microenvironment and increase the infiltration and activation of immune cells, thus improving immunotherapy responsiveness. High doses of radiotherapy may inhibit the function of immune cells and reduce the effectiveness of immunotherapy (69).

Radiotherapy doses can be divided into low-dose radiotherapy (0.1–1 Gy), high-dose radiotherapy (8 Gy and above), and standard clinical doses (1.8-2.2 Gy) (71). Low dose radiotherapy can increase adaptive immune resistance mechanisms within the tumor, including mobilization of dendritic cells and activation of effector CD4+ and CD8+ T cells. These cells exhibit cytotoxic transcriptional programs in which NKG2D expression plays an important role (72). High doses of radiotherapy cause tumor cell death and DNA damage, which releases immunogenic substances and activates the immune system. High doses of radiotherapy lead to an increase in the number and function of regulatory T cells (TreGs), which can suppress the immune response (73). High-dose radiotherapy can alter the tumor microenvironment, and high-dose radiotherapy can alter the cellular and molecular composition surrounding the tumor, including tumor-associated fibroblasts and immune cells (74). High-dose radiotherapy can increase the immunogen released by tumor cells, thereby enhancing the immune system’s recognition and attack of the tumor. This enhanced immunogenicity may help improve the therapeutic effectiveness of radiation therapy (64). Standard clinical doses of radiation therapy are often used to treat a variety of solid tumors. This segmented treatment lasts for several weeks to minimize toxicity to normal tissue. At the same time, the lymphocytes are rapidly cleared, reducing the effectiveness of the immune response (71).

The effective dose of immune cells is the equivalent uniform dose applied to immune cells during radiation therapy. It is estimated by calculating the amount of radiation received by the immune cells circulating in the blood (75). There is a direct correlation between EDIC and lymphocyte nadir, and both EDIC and lymphocyte nadir can predict long-term survival. The accuracy of EDIC in predicting overall survival (OS) was comparable to that of lymphocyte NAdir. The results showed that EDIC was an independent predictor of lymphocyte trough, progression-free survival (PFS) and OS. Therefore, EDIC can be used as a surrogate marker for lymphocyte trough and OS in patients with restricted small-cell lung (76). For patients with non-small cell lung cancer, EDIC is associated with progressive disease-free survival (PFS) and overall survival (OS) in patients with NSCLC. Patients with lower EDIC have longer PFS and OS. EDIC has also been associated with severe lymphopenia and treatment-related toxicity (77).

**Figure 4:**
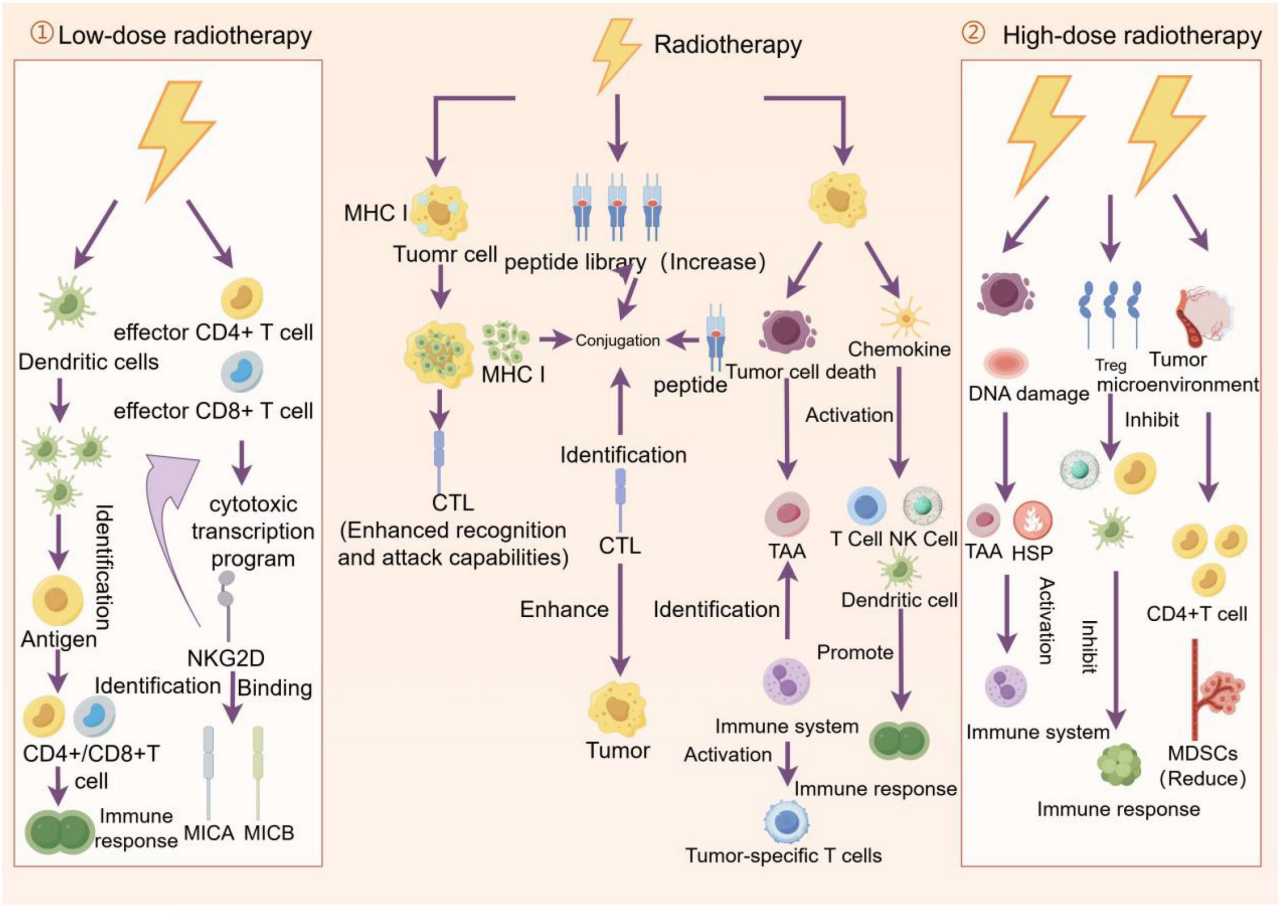
Effects of low - and high-dose radiation therapy on tumor cells and the immune system. Low-dose radiation therapy promotes recognition of tumor antigens by dendritic cells and activates effector CD4+ and CD8+ T cells, thereby enhancing the immune response. Standard radiotherapy enhances the ability of cytotoxic T lymphocytes (CTLS) to recognize and attack tumors by increasing the library of peptides displayed by MHC I molecules on the surface of tumor cells, while promoting tumor cell death and immune response. High-dose radiotherapy, on the other hand, triggers tumor cell death and DNA damage, activates dendritic cells and T cells, further promoting the immune response, while inhibiting regulatory T cells (TreGs) and myeloid suppressor cells (MDSCs), reducing immunosuppression and thus fighting the tumor more effectively.

The application time of radiotherapy and immunotherapy should be comprehensively considered according to the specific conditions. In some cases, radiation therapy may be administered before immunotherapy to reduce tumor volume and increase the effectiveness of immunotherapy. In other cases, radiation may be given after immunotherapy to control residual tumor cells (78). Applying radiation and immunotherapy together may improve the effectiveness of the treatment. Some studies have found that applying both PD-1 and CTLA-4 immune checkpoint inhibitors and radiation therapy at the same time can improve patient survival. The mechanism of this combination treatment may involve radiation enhancing the immune system’s response, making immunotherapy more effective. However, specific treatment regimens and effects may vary depending on individual differences and tumor type (79) (**Figure 4**).

The delayed effects of radiotherapy mainly apply to craniocerebral radiotherapy. Craniocerebral radiotherapy can change the permeability of the blood-brain barrier, which not only helps to enhance the local therapeutic effect, but also promotes the migration of immune cells to the central nervous system in the systemic immune response. Specifically, craniocerebral radiotherapy is able to temporarily open the blood-brain barrier by increasing the expression of vascular endothelial growth factor (VEGF) and matrix metalloproteinases (MMPs), thus allowing systemic therapies to more easily enter the brain tissue (80).

## 6 Stereotactic external radiation therapy/stereotactic accelerated radiation therapy with immunosuppression

SBRT uses a high-dose beam of radiation to precisely irradiate the tumor to maximize the destruction of tumor cells and minimize damage to surrounding normal tissue. SBRT typically requires fewer treatments and a higher dose per treatment than conventional radiation therapy. This treatment is suitable for small tumors or tumors that cannot be removed surgically and can provide a higher therapeutic effect (81). SBRT can induce immunogenic cell death (ICD) of tumor cells by promoting the release of tumor-associated antigen (TAA) and Major histocompatibility Complex I (MHC I), which triggers antigen presentation. In addition, SBRT can directly stimulate dendritic cell (DC) maturation and CD8+ cell infiltration into the tumor microenvironment (TME). In contrast, conventional radiotherapy induces an increase in immunosuppressive cells such as regulatory T cells (Tregs), M2 type macrophages, and myelopathic cells (82). After SABR treatment, tumor-derived extracellular vesicles (EVS) released by tumor cells can suppress the anti-tumor immune response. The immunosuppressive molecules in EVs can interfere with the activation of T cells and the killing effect of tumor cells, thus promoting the occurrence of distant metastasis. The expression of PD-L1 and B7-H3 in EVs increases after SABR treatment. The upregulation of these immune checkpoint molecules may be in response to radiation therapy (83). In conclusion, with more research on SBRT, we expect to discover more strategies to enhance its efficacy. For example, combining immunotherapies, such as PD-1/PD-L1 inhibitors, can neutralize the immunosuppressive molecules in EVs, thereby restoring the anti-tumor immune response. In addition, through gene-editing technologies such as CRISPR-Cas9 and others, we can more precisely regulate the way tumor cells die, so that they release more molecules with immunostimulatory effects, further enhancing the efficacy of SBRT.

Radiation from SBRT to tumor cells can induce the expression of IDO and PD-L1, both of which have been implicated in immunosuppression. Compared with other studies, the upregulation effect of SBRT on IDO expression was weaker. This suggests that SBRT may induce immunosuppression primarily by activating the PD-1/PD-L1 pathway, while IDO plays a relatively minor role (84). The study found that absolute monoaminic acid (kynurenine) levels of IDO increased significantly after radiotherapy in 3DCRT patients, while there was no significant change in SBRT patients. This suggests that SBRT may have a weaker inhibitory effect on the immune system. The study also found that IDO activity in SBRT patients was not associated with survival outcomes, while IDO activity in 3DCRT patients was associated with poorer survival outcomes. These results suggest that SBRT may have a less inhibitory effect on the immune system than 3DCRT, which may be related to the fact that SBRT is treated in a more precise and targeted manner (85).

**Figure 5:**
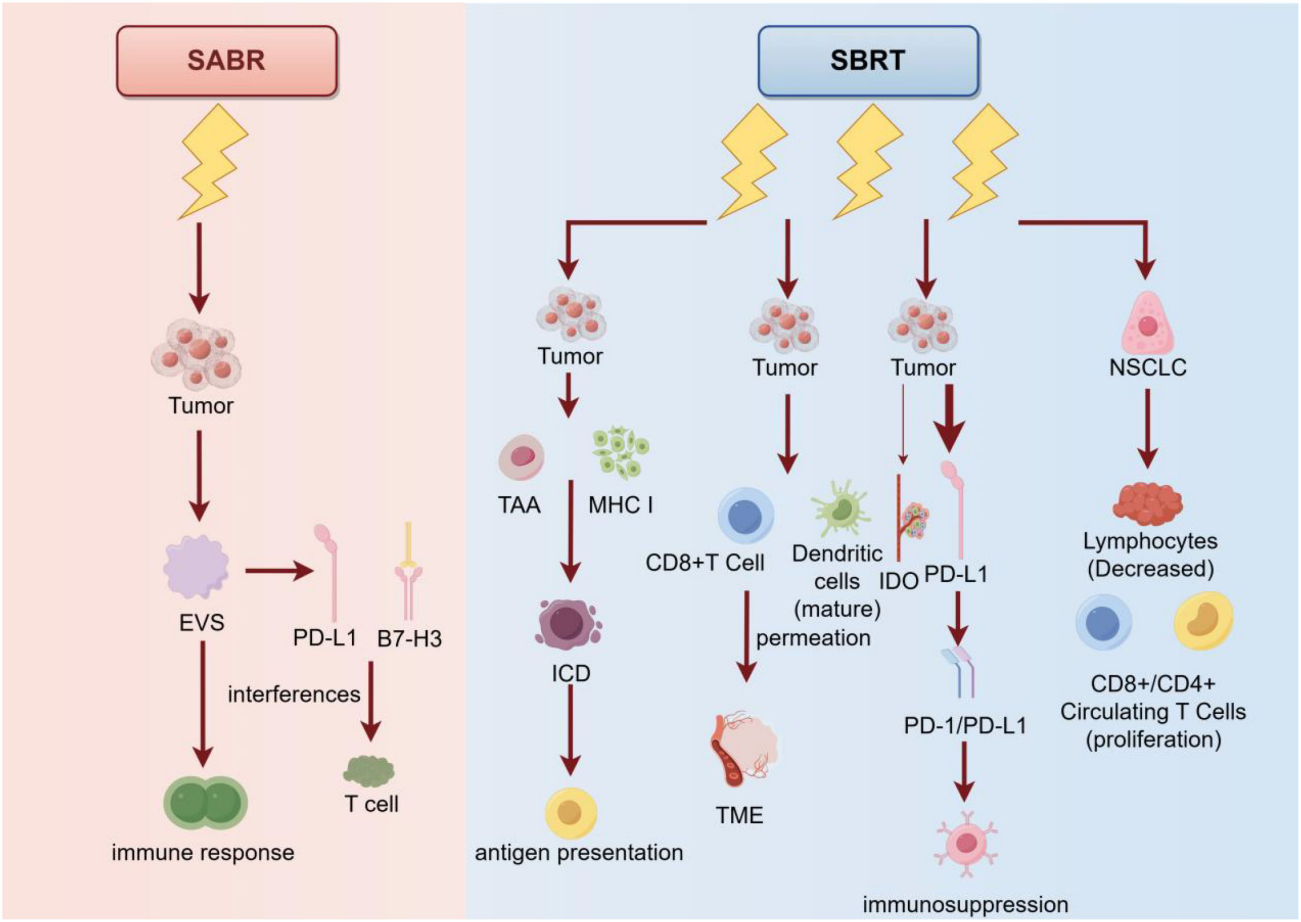
Principles, mechanisms, and their use in cancer treatment of the two therapies SABR and SBRT. SABR suppresses the immune response by acting directly on tumor cells and inducing them to release exosomes (EVS), which interfere with T cells via PD-L1 and B7-H3 molecules. In contrast, SBRT not only similarly acts on tumor cells, promoting tumor antigen (TAA) via MHC I molecule presentation and enhancing immunocytotoxicity (ICD), but also promotes dendritic cell maturation and T cell activation, inhibits immunosuppression via PD-L1 and IDO pathways, Which in turn promotes the proliferation of CD8+ and CD4+ circulating T cells. In addition, SBRT reduces the number of lymphocytes, which enhances the immune response to non-small cell lung cancer (NSCLC). Overall, SBRT was more effective than SABR in enhancing the anti-tumor immune response.

Studies have shown that the dose of lymphocytes before stereotactic radiotherapy (SBRT) is negatively correlated with overall survival in patients with non-small cell lung cancer (NSCLC), i.e., the lower the dose of lymphocytes, the shorter the overall survival (11). SBRT treatment can lead to transient lymphocytopenia, but at the same time increases the proliferation of CD8+ and CD4+ circulating T cells (86). SBRT therapy can enhance the immune response and improve patient survival by increasing the number and activity of tumor-specific CD8+ T cells. One study observed a temporary increase in the number of tumor-specific CD8+ T cells 5 to 8 days after SBRT treatment in patients with non-small cell lung cancer, with inhibitory regulatory T cells (Treg cells) dominating for the next 10 to 16 days. This suggests that there may be a change in the immune response for some time after SBRT treatment. Therefore, the ideal time interval would be to combine SBRT-induced immune sensitivity with PD-1/PD-L1 inhibitor-induced immune activation (87). PD-1/PD-L1 inhibitors are a type of immunotherapy that neutralizes inhibitory signals, thereby restoring the antitumor activity of T cells. By using the two in combination, one can expect to neutralize the inhibition of Treg cells with PD-1/PD-L1 inhibitors for some time after SBRT treatment, thereby further enhancing the immune response and improving the effectiveness of the treatment (**Figure 5**).

## 7 Proton therapy versus photon therapy

Proton therapy is a radiation therapy technique that uses high-energy proton beams to treat tumors. Proton therapy has a better dose distribution and fewer side effects than traditional photon radiation therapy. Proton beams can accurately deliver higher doses to the tumor area while minimizing damage to surrounding normal tissue (88). Photon therapy, as one of the most widely used radiation therapies today, mainly treats cancer by utilizing high-energy X-rays or gamma rays. The process involves accurately projecting beams of high-energy photons onto the tumor area, effectively destroying cancer cells or inhibiting their further growth. Photon therapy can use different techniques to adjust the distribution of radiation doses according to the shape and location of the tumor to minimize damage to surrounding normal tissue (89).

There are differences in biological responses to some endpoints between proton therapy and photon therapy. Proton therapy differs from photon therapy in terms of DNA damage and repair, angiogenesis, and cell migration. These differences can have an impact on treatment effectiveness and side effects (90). A study of proton therapy versus photon therapy in patients with esophageal cancer found that proton therapy showed some advantages in patients with esophageal cancer. First, proton therapy can provide a more precise dose distribution, which can better protect the surrounding normal tissue and reduce the occurrence of side effects. Second, proton therapy is able to deliver a larger dose of radiation directly to the tumor site, thus enhancing the treatment’s effectiveness. Finally, proton therapy can not only accurately deliver high-dose radiation to the tumor site, but also significantly reduce radiation exposure to key organs such as heart and lung, thus effectively reducing the risk of possible complications after treatment (91). Studies of proton therapy and photon therapy in patients with stage III non-small cell lung cancer (NSCLC) have found that proton therapy can reduce the incidence of severe lymphocytopenia and anemia, and perform better in the assessment of physical condition after radiotherapy (92).

FLASH radiotherapy is a type of ultra-high dose rate radiation therapy that has a dose rate several orders of magnitude higher than conventional clinical radiotherapy. FLASH radiotherapy triggers a phenomenon known as the FLASH effect, in which ultra-high dose rate radiation reduces normal tissue toxicity common to conventional radiotherapy while still maintaining local tumor control (93). A study using a proton beam as a radiation source for FLASH radiation therapy, by comparing the effects of FLASH radiation therapy with conventional radiation therapy in a lung cancer model in mice, found that FLASH radiation therapy could better control tumor growth and was able to increase immune cell infiltration and enhance immune response, thereby improving treatment outcomes (94). Both conventional and FLASH dose rate proton radiotherapy were able to induce an effective lymphocyte immune response. Significant increases in tumor-infiltrating lymphocytes were observed in the tumor microenvironment, including regulatory T cells (TREGs), tissue-resident memory cytotoxic T cells (CD8+ TRM T cells), natural killer cells, and B cells. This suggests that proton radiotherapy with both conventional and FLASH dose rates was able to activate an adaptive immune response against the tumor (95).

## 8 Future perspectives

Although significant progress has been made in the integration of radiotherapy and immunotherapy, several critical challenges and opportunities for further development remain. In terms of individualized treatment strategies, while key biomarkers such as NLR and IDO have been identified, the translation of these markers into personalized therapeutic decision-making—including dose optimization and management of treatment-related toxicities through precision medicine—remains to be fully elucidated. Regarding long-term efficacy and safety, current evidence largely reflects short-term outcomes, with limited experimental and clinical data on the prolonged effects of combined modalities across different tumor types. For instance, RT-induced lymphopenia may compromise systemic immune function, potentially influencing tumor recurrence and metastasis, yet this phenomenon remains poorly characterized in the context of long-term patient outcomes. While technological advancements such as proton beam therapy offer promising new avenues for enhancing therapeutic precision and immune activation, their integration with immunotherapeutic approaches has not been comprehensively evaluated. To address these challenges, future research should adopt a multi-dimensional approach: deepening personalized medicine by tailoring therapeutic regimens based on patients’ genetic profiles, immune status, and biomarker expression; exploring novel combination strategies that incorporate emerging immune checkpoint inhibitors such as LAG-3, TIM-3, and TIGIT into existing therapeutic frameworks; strengthening long-term follow-up studies to systematically assess post-treatment health trajectories in treated populations; promoting interdisciplinary collaboration among oncology, immunology, radiation physics, and other relevant disciplines to foster innovation and knowledge integration; and actively evaluating the clinical potential of cutting-edge technologies—including proton therapy and CAR-T cell therapy—in combination with conventional treatment modalities. These efforts are essential to unlock the full therapeutic potential of RT–IT combinations and to pave the way for more effective and durable cancer treatments.

## 9 Conclusion

The combination of radiotherapy and immunotherapy represents an important breakthrough in the field of cancer treatment. Radiation therapy not only exerts anti-tumor effects by directly killing tumor cells, but also activates the systemic anti-tumor immune response by releasing tumor-associated antigens, reshaping tumor microenvironment, and enhancing immune cell infiltration. Immunotherapy, especially immune checkpoint inhibitors (such as PD-1/PD-L1 and CTLA-4 inhibitors), further enhances the efficacy of radiotherapy by lifting the tumor’s suppression of the immune system. Clinical studies have shown that the combined application of radiotherapy with immunotherapy has shown significant synergies in multiple cancer types, especially in refractory tumors such as non-small cell lung cancer, melanoma and liver cancer, significantly improving patient survival and quality of life.

However, the combined application of radiotherapy and immunotherapy still faces many challenges. First, the effects of dose, frit and timing of radiotherapy on immune regulation are not fully understood, and different doses and frit regimens may have radically different effects on the immune system. Second, combination therapy may increase the incidence of immune-related adverse effects (such as immune pneumonia, hepatitis, etc.), especially after high doses of radiotherapy. In addition, the heterogeneity of the tumor microenvironment may also affect the efficacy of combination therapy, and some patients may develop resistance to combination therapy.
